# CXCL16/CXCR6 chemokine signaling mediates breast cancer progression by pERK1/2-dependent mechanisms

**DOI:** 10.18632/oncotarget.3690

**Published:** 2015-03-29

**Authors:** Gang Xiao, Xiumin Wang, Jinglong Wang, Lidong Zu, Guangcun Cheng, Mingang Hao, Xueqing Sun, Yunjing Xue, Jinsong Lu, Jianhua Wang

**Affiliations:** ^1^ Department of Biochemistry and Molecular Cell Biology, Shanghai Key Laboratory of Tumor Microenvironment and Inflammation, Shanghai Jiao Tong University School of Medicine, Shanghai, China; ^2^ Comprehensive Breast Health Center, Renji Hospital, Shanghai, China; ^3^ Cancer Institute, Fudan University Shanghai Cancer Center, Shanghai, China

**Keywords:** CXCL16/CXCR6, breast cancer, ERK1/2, RhoA

## Abstract

Our previous studies demonstrate that CXCL6/CXCR6 chemokine axis induces prostate cancer progression by the AKT/mTOR signaling pathway; however, its role and mechanisms underlying invasiveness and metastasis of breast cancer are yet to be elucidated. In this investigation, CXCR6 protein expression was examined using high-density tissue microarrays and immunohistochemistry. Expression of CXCR6 shows a higher epithelial staining in breast cancer nest site and metastatic lymph node than the normal breast tissue, suggesting that CXCR6 may be involved in breast cancer (BC) development. *In vitro* and *in vivo* experiments indicate that overexpression of CXCR6 in BC cells has a marked effect on increasing cell migration, invasion and metastasis. In contrast, reduction of CXCR6 expression by shRNAs in these cells greatly reduce its invasion and metastasis ability. Mechanistic analyses show that CXCL16/CXCR6 chemokine axis is capable of modulating activation of RhoA through activating ERK1/2 signaling pathway, which then inhibits the activity of cofilin, thereby enhancing the stability of F-actin, responsible for invasiveness and metastasis of BC.

Taken together, our data shows for the first time that the CXCR6 / ERK1/2/ RhoA / cofilin /F-actin pathway plays a central role in the development of BC. Targeting the signaling pathway may prove beneficial to prevent metastasis and provide a more effective therapeutic strategy for BC.

## INTRODUCTION

Chemokines are a family of small (8–14 kDa), mostly basic, heparin-binding cytokines that primarily induce directed migration of various types of leukocytes through interactions with a group of seven transmembrane G protein-coupled receptors (GPCR) [1]. To date, over 50 chemokines and 20 chemokine receptors have been identified, and are grouped into four categories (C, CC, CXC and CX3C) according to the location of the main cysteine residues near the N terminal of these proteins [1]. The chemokine receptor system extends to most human neoplastic cells and was found to be altered dramatically in neoplastic tissue, particularly at the leading edge of invasion [2]. CXCL16 is one of the only two known plasma membrane chemokines. In a previous study, CXCL16 was found not only in immune cells, but also expressed constitutively in cancer cells of different origins [3-6]. CXCR6 was identified as a cognate receptor of CXCL16 usually expressed by peripheral blood leukocytes [7]. Interestingly, CXCR6 also has been shown to be present in prostate tissues and in the marrow [8].

Our previous study and other studies have proposed an important role for CXCL16/CXCR6 chemokine axis in the metastasis of different tumors including prostate, liver, ovarian cancers [4, 9, 10]. Through a cytokine antibody array, Lu et al. reported that CXCL16 protein production was increased in aggressive PCa cells compared to the less aggressive PCa cells or benign prostate cells [11]. These observations raise the possibility that CXCL16/CXCR6 interactions may be important for tumor invasion and metastasis, as was demonstrated for CXCL12/CXCR4 chemokine axis [4, 12]. Recently, Cheng's studies indicate that different invasive breast cancer (BC) cell lines express CXCR6 at different levels, positively correlated with its invasive ability [13]; however, whether CXCR6 plays a role in BC invasion and metastasis is still kept unclear.

BC is the leading cause of cancer in women worldwide [14]. The molecular basis of the complex biochemical processes involved in BC, including tumor growth and metastasis, is becoming increasingly understood. Poor prognosis and high mortality are explicitly correlated with tumor invasion and metastasis. Tumor cell invasion and metastasis are a complex process; however, the increasing evidence has recently shown that the chemokines and their receptors are involved [1, 2]. Therefore, exploring the mechanism of CXCL16/CXCR6 chemokine axis may prove beneficial to prevent metastasis and provide a more effective therapeutic strategy for BC.

Here we found that CXCR6 expression was significantly higher in BC tissues and the metastatic lymph nodes than in normal breast tissues. Moreover, we found that CXCR6 increased cell migration, invasion and metastasis, associated with increased phosphorylation of p42/44MAPK (ERK1/2) proteins. In contrast, reduction of CXCR6 expression by shRNAs in these cells greatly reduced these effects. Notable, we found that the enhanced activity of ERK1/2 pathway was able to activate RhoA, one of members of the RhoGTPase family. The effect led to inhibiting the activity of cofilin, thereby enhancing the stability of F-actin, associated with the invasiveness and metastasis of BC.

## RESULTS

### Expression of CXCR6 in BC tissues and cell lines

**Figure 1:**
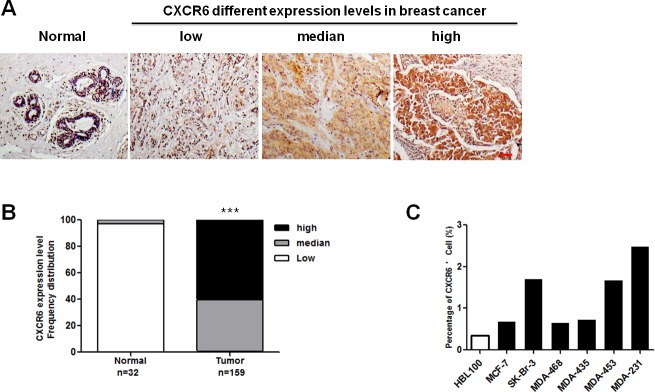
CXCR6 is higher expressed in BC tissues and cell lines than in the normal controls **A**) CXCR6 immunohistochemical staining in a human BC tissue microarray. Representative microscopic images of normal breast, and BC tissues stained with an anti-human CXCR6 antibody (1:200, Epitomics). Scale bar, 50 μ m. **B**) Chi-square test was used to analysis the CXCR6 expression differences in tumor and normal tissues. Based on staining intensity, the samples were classified into three groups with increasing staining intensity from the weakest (low) to the strongest (high). CXCR6 expression in BC tissues is significantly higher than in normal breast tissues (including para-carcinoma tissues). **C**) CXCR6 positive expression rate of normal mammary epithelial cell HBL100 and variety of BC cell lines was analyzed by flow cytometry. Cells incubated with allophycocyanin (APC)-conjugated monoclonal anti-human CXCR6 (R&D, Cat, FAB699A) for 30-45 minutes at 2-8°C were detected by flow cytometry for CXCR6 expression. The histogram shows the ratio of CXCR6 positive cells in the presented cell lines.

Emerging evidence suggests that CXCR6 expression is responsible for progression in multiple cancers [5, 15, 16]. However, its role in BC development still remains unclear. In this regard, high-density tissue microarrays were stained with an anti-human CXCR6 antibody from clinical samples obtained from a cohort of about 300 patients. Representative images are shown in Figure 1A and Supplementary Figure 1A, demonstrating that CXCR6 expression increases in the primary tumor and metastatic lymph node, whereas normal epithelium demonstrates weak cytoplasm staining. Quantitative analysis confirms that CXCR6 expression is increased in tumor (Figure 1B, Supplementary Figure 1B and Supplementary Table 1). Similar results are observed in malignant BC cell lines by flow cytometric analysis (Figure 1C). Therefore these findings suggest that CXCR6 expression may be correlated with BC progression.

### CXCR6 promotes BC cell migration and invasion

**Figure 2:**
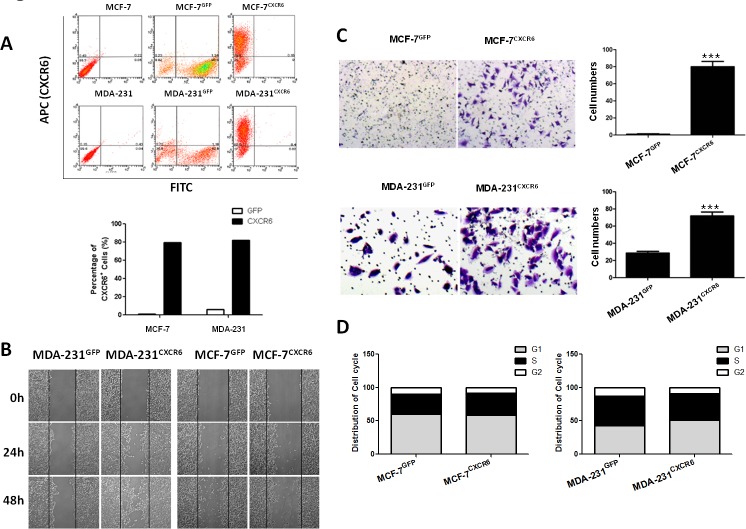
CXCR6 promotes BC cells migration and invasion **A**) CXCR6 was overexpressed in MCF-7 and MDA-231 BC cells by lentiviral system. CXCR6 -positive cells were then sorted by flow cytometry. Histogram displays the ratio of CXCR6 positive expression of MCF-7^CXCR6^, MDA-231^CXCR6^ and their respective control cells. **B**) Scratch wound healing assay shows that overexpression of CXCR6 enhanced cell migration in MDA-231 cells, but not very significant in MCF-7 cells. **C**) Matrigel invasion assay showed that overexpression of CXCR6 significantly enhanced cell invasion in both MCF-7 and MDA-231 cells (^***^P < 0.001). Experiments were performed 3 times; representative images of invaded cells are shown. Mean ± SD. **D**) Cytometry analysis showed CXCR6 had no effect on cell cycle.

A critical feature of BC is highly aggressive behavior in distant metastasis sites including lymph node, bone, lung, and live, *et al*. [17, 18]. To further explore the role of CXCR6 in BC progression, lentiviral vectors were utilized to overexpress CXCR6 in MCF-7 and MDA-231 cells (termed as MCF-7^CXCR6^ and MDA-231^CXCR6^), or reduce CXCR6 expression mediated by shRNAs (termed as MDA-231^shCXCR6^). After clone selection, individual clones were selected and pooled. Figure 2A showed that overexpression of CXCR6 in both cells was confirmed by flow cytometry.

Once individual tumor cells are bound to the endothelium, they must invade through the extracellular matrix in order to establish a metastasis. The ability of CXCR6 to regulate migration and invasion was assessed by scratch healing assay and using reconstituted extracellular matrices in porous culture chambers. The results show that MCF-7^CXCR6^ and MDA-231^CXCR6^ cells gradually closed the wound at 48 hour after scratch, whereas the respective control cells didn't gain the effect (Figure 2B). In line with this finding, the invasive capacity was greatly increased in MCF-7^CXCR6^ and MDA-231^CXCR6^ cells (Figure 2C).

To exclude the potential effect of cell proliferation on migration and invasion, we next assayed cell cycle distribution by flow cytometric analysis. As shown in Figure 2D, we found that overexpression of CXCR6 did not apparently affect cell cycle progression (Supplementary Figure 2). Taken together, these results confirm that CXCR6 plays a role in migration and invasion of BC cells.

### Activation of ERK1/2 signaling by CXCR6 promotes BC cell invasion

CXCR6, as a G protein-coupled receptor, can be combined with its cognate ligand CXCL16 to activate downstream signaling pathways. Previously, we demonstrated that CXCL16/CXCR6 chemokine axis induces prostate cancer progression by the AKT/mTOR signaling pathway [4].

**Figure 3:**
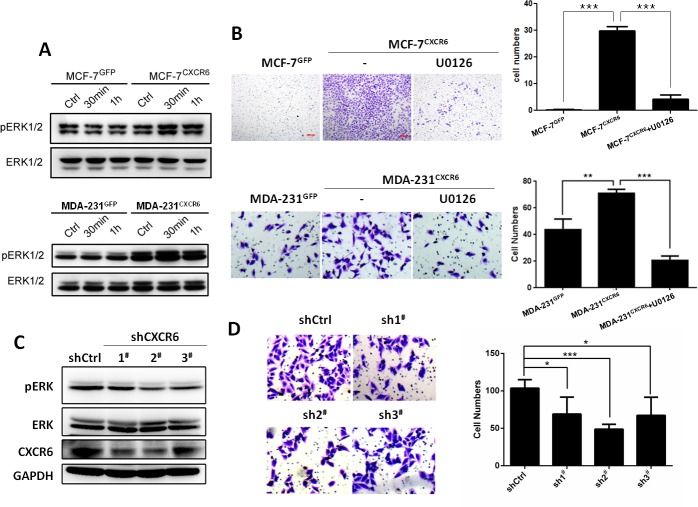
CXCL16/CXCR6 chemokine axis promotes cell invasion by ERK1/2 signalling dependant manner **A**) BC cells treated with CXCL16 (100 ng/ml) for indicated time (0, 30, 60 minutes), the pERK1/2 pathway was significantly activated in MCF-7^CXCR6^ and MDA-231^CXCR6^ cells. The experiments were performed three times. **B**) Matrigel invasion assay showed when block the pERK1/2 pathway with U0126, the CXCR6 induced cell invasion capacities reduced in both MCF-7 and MDA-231 (^**^P < 0.01, ^***^P < 0.001). Experiments were performed 3 times; representative images of invaded cells are shown. Mean ± SD. **C**) CXCR6 knockdown by shRNA1^#^, 2^#^, 3^#^ can attenuate the pERK1/2 pathway in 231^CXCR6^ cells. The experiments were performed three times. **D**) Matrigel invasion assay showed CXCR6 knockdown by shRNA1^#^, 2^#^, 3^#^ inhibited cell invasiveness (^*^P < 0.05, ^***^P < 0.001). Experiments were performed 3 times; representative images of invaded cells are shown. Mean ± SD.

Next, we determine which pathway is involved in activation of CXCR6 by CXCL16 stimulation, associated with BC cell invasion. As shown in Figure 3A, Western blot analyses show that stimulation of CXCL16 can apparently activate ERK1/2 pathway by a time-dependent manner in MCF-7^CXCR6^ and MDA-231^CXCR6^ cells compared with the respective control cells. To further confirm that ERK1/2 pathway is responsible for CXCR6-induced cell invasion, we blocked ERK1/2 activation by U0126, a specific inhibitor of MEK1/2 kinase in upstream of ERK1/2 signaling. The result shows that U0126 is able to greatly block the increasing invasion caused by CXCR6 overexpression in MCF-7 and MDA-231cells (Figure 3B). In contrast, CXCR6 knockdown in MDA-231^CXCR6^ cells by shRNAs, ERK1/2 activity is greatly inhibited, followed by the reduction of cell invasion (Figure 3C and 3D). Taken together, these data suggest that CXCL16 stimulation through CXCR6 activates ERK1/2 signaling pathway in BC cells, responsible for cell invasion.

### CXCR6 modulates formation of stress fibers in an ERK1/2 signaling dependent manner

Factors that affect cellular motility or invasion include cellular polarity, cytoskeleton organization, and transduction of signals from the outside environment [19-21]. To investigate the state of cell polarity, we initially detected the expression of E-cadherin and vimentin. Western analysis show that cells treated with CXCL16 at different time points, both E-cadherin and vimentin are no significant changes (Supplementary Figure 3), suggesting that CXCL16/CXCR6 signaling is irresponsible for EMT, potentially involved in BC invasion.

In this regard, to explore potential downstream targets induced by CXCR6, we analyzed the genome-wide transcriptome profile of MCF-7^CXCR6^, MDA-231^CXCR6^ and their respective control cells by Agilent Whole Human Genome Microarrays. The microarray data set has been deposited in the GEO database (http://www.ncbi.nlm.nih.gov/geo/query/acc.cgi?acc=GSE60484). According to fold-change (×2.0) screening between CXCR6 overexpression and its respective control, 49 genes exhibit consistent change trend in both MDA-231^CXCR6^ νs MDA-231^GFP^ and MCF-7^CXCR6^ νs MCF-7^GFP^ cell pairs, in which 35 genes are down-regulated and 14 genes are up-regulated (Supplementary Figure 4A). Supplementary Figure 4C and 4D show the cluster mapping of the picked up 49 genes might participate in invasion or metastasis by the MeV 4.6 microarray analysis software. Gene ontology (GO) cellular component analysis shows the cytoskeleton is the secondly largest fold enrichment GO term (Supplementary Figure 4B), which suggesting that CXCR6 induced cell migration and invasion may involve the changes in cytoskeleton. All those genes with more than 2 fold change are showed in Supplementary Figure 5A and B. Real-time PCR was used to further confirm the above microarray results as shown in Supplementary Figure 5C.

**Figure 4:**
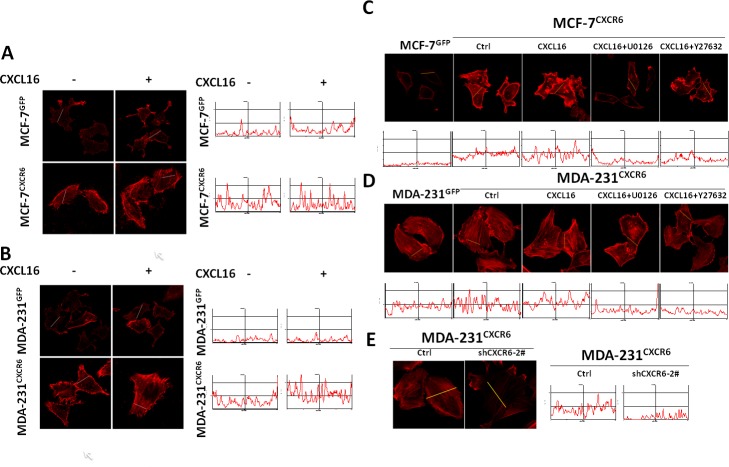
CXCL16/CXCR6 chemokine axis promotes stress fibres assembly through ERK1/2 pathway **A**) and **B**) CXCL16/CXCR6 chemokine axis promotes F-actin assembly. Cells treated with or without CXCL16 for 5 minutes, F-actin was visualized by Rhodamine-phalloidin under a Zeiss LSM 710 Laser Scanning Confocal Microscopy. The fluorescence intensity was quantified using Image Pro Plus 6.0 software. **C**) and **D**) ERK1/2 pathway involves in CXCL16/CXCR6 induced F-actin assembly. Pretreatment of cells with or without U0126 or Y27632 for 30 minutes, then continue treatment with CXCL16 for 5 minutes, F-actin was visualized as previous mentioned.

Among the genes markedly regulated by CXCR6 overexpression in MDA-231 and MCF-7 cells, we focused on ACTG2 (Supplementary Figure 4D and 5C), which is the monomeric form of F-actin. F-actin as actin stress fibers is one of the major cytoskeletal structures, closely responsible for the elevated cellular motility and invasion[22]. Here, we firstly examined the cytoskeletal morphology by immunofluorescence staining of F-actin. The result shows that actin stress fibers are somehow increased in MCF-7^CXCR6^ and MDA-231^CXCR6^ cells compared with the respective controls even in absence of CXCL16 stimulation (Figure 4A and 4B). However, stimulation of CXCL16 induces more intense expression of actin stress fibers in MCF-7^CXCR6^ and MDA-231^CXCR6^ cells than in the respective controls (Figure 4A and 4B). Quantitative analyses confirm the above observations (Figure 4A and 4B).

As previously reported, Rho/ROCK activation could lead to actomyosin-based contractility and actin cytoskeletal reorganization through indirectly phosphorylating cofilin and MLC, which could alter the integrity of TJ(tight junction) [23, 24]. To elucidate the mechanistic roles of CXCR6 in cytoskeleton organization, inhibitors of ERK1/2 and Rho/ROCK pathways were next used. Immunofluorescence assays show that U0126 is able to apparently block the intense expression of actin stress fibers caused by CXCR6 overexpression in both cells in presence of CXCL16 stimulation. Similar findings are observed in Y27632 treatment, an inhibitor of the Rho/ROCK pathway (Figure 4C and 4D). Quantitative analyses confirm the above observations (Figure 4C, and 4D). In contrast, reduction of CXCR6 expression by shRNAs in MDA-231^CXCR6^ cells potentially decreased the intense expression of actin stress fibers compared with the respective control (Figure 4E). To further explore the effect, we found that the phosphorylation levels of cofilin are up-regulated by CXCL16 in dose-dependent manner, suggesting that cofilin is involved in regulating process through CXCL16/CXCR6 chemokine axis. In addition, FAK is activated by CXCL16 stimulation, which may partly contributed to the enhanced cell migration and invasion (Supplementary Figure 6A).

Overall, these results suggest that CXCL16 stimulation through CXCR6 may modulate formation of stress fibers in an ERK1/2/RhoA/cofilin signaling dependent manner.

### RhoA/cofilin regulated by ERK 1/2 signaling contributes to the formation of stress fibers

The above findings also suggest that the formation of stress fibers is regulated by the small GTP-binding protein Rho. The small GTPase Rho is inactive in its guanosine diphosphate (GDP)- bound form and active in its guanosine triphosphate (GTP)- bound form, which binds to specific targets that mediate its biological functions including the formation of actin stress fibers[25, 26].

**Figure 5:**
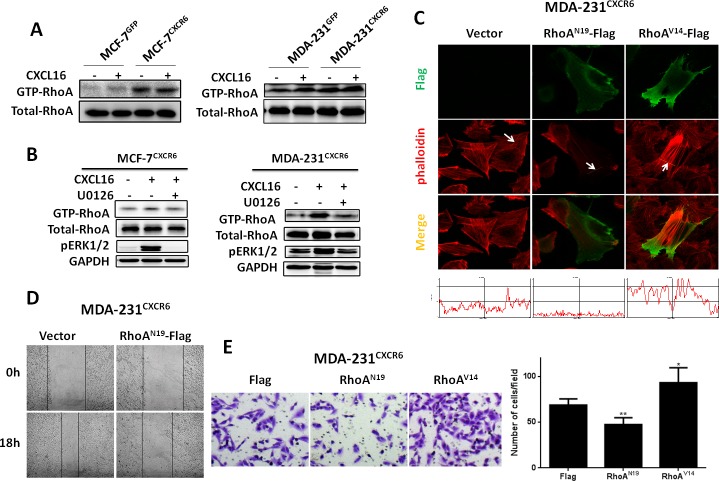
RhoA GTPase mediates F-actin assembly and BC cell migration and invasion **A**) CXCL16/CXCR6 chemokine axis promotes the activation of RhoA. **B**) Pretreatment of cells with or without U0126 for 30 minutes, then continue treatment with CXCL16 for 5 minutes, GTP-RhoA was isolated using active Rho pull-down and detect kit, total RhoA in the total cell lysates and GTP-RhoA were detected by Western- blot analysis. **C**) MDA-231^CXCR6^ cells were transfected with RhoA^V14^-flag or RhoA^N19^-flag plasmids using Lipofectamine 2000, 24 hours later, cells were fixed for immunofluorescence assay, F-actin (Red) and flag (Green) were detected under a Zeiss LSM 710 Laser Scanning Confocal Microscopy. **D**) Inactivation of RhoA in BC cells reduces migration properties. MDA-231^CXCR6^ cells were transfected with RhoA^N19^-flag or control plasmids for wound healing assay. Representative images at time points 0 and 18 hours. **E**) Matrigel invasion assay shows RhoA activity positively correlated with cell invasion. Experiments were performed 3 times; representative images of invaded cells are shown. Mean ± SD.

Active RhoA pull-down assays initially show that GTP-RhoA expression is increased in MCF-7 and MDA-231 control cells when treated with CXCL16 for 5 min (Figure 5A). Notable, the result also indicates that CXCR6 overexpression induces more active GTP-RhoA expression in both cells than in the respective controls, regardless of CXCL16 treatment (Figure 5A). Based on the findings, we next explore whether the activation of the ERK1/2 pathway is able to regulate RhoA activity. As shown in Figure. 5B, blocking the ERK1/2 pathway with U0126 significantly reduced the activation of RhoA in MCF-7^CXCR6^ and MDA-231^CXCR6^ cells (Figure 5B). These findings suggest that CXCL16/CXCR6 signaling pathway regulates the activity of RhoA in an ERK1/2 dependent manner.

To further explore the role of RhoA in the formation of stress fibers, we transfected the constitutively active RhoA vector RhoA^V14^ or dominant negative RhoA vector RhoA^N19^ in MDA-231^CXCR6^ cells, either of which was tagged with three Flag-tags at C terminal. Immunofluorescence assays show that more intense F-actin formation is observed in RhoA^V14^-trensfected MDA-231^CXCR6^ cells than the control. In contrast, RhoA^N19^-trensfected MDA-231^CXCR6^ cells induce less F-actin formation (Figure 5C). Quantitative analyses confirm the above observations (Figure 5C). Moreover, wound healing assay shows that RhoA^N19^-transfected MDA-231^CXCR6^ cells significantly reduce BC cell migration and invasion, while RhoA^V14^-transfected MDA-231^CXCR6^ cells significantly enhance BC cell invasion compared with the control (Figure 5D-E). These findings suggest that the activated RhoA promotes BC cell invasion and migration, and vice versa.

Given CXCL16/CXCR6 chemokine axis promotes RhoA activation and the formation of F-actin is regulated by activity of RhoA, we transfected dominant negative RhoA^N19^ into MDA-231^CXCR6^ cells. Supplementary Figure 6B shows that phosphorylation of cofilin is inhibited by DN-RhoA^N19^. These findings suggest that the stress fibers formation by CXCL16/CXCR6 chemokine axis is potentially responsible for RhoA/cofilin pathway, which is capable of enhancing stability of stress fibers.

Taken together, these data suggest that CXCR6 expression induces the increased formation of stress fibers in BC cells, dependent on the RhoA/cofilin pathway in an ERK1/2 dependent manner, which promotes BC cell migration and invasion.

### Effect of targeting CXCR6 expression on tumor metastasis *in vivo*

**Figure 6:**
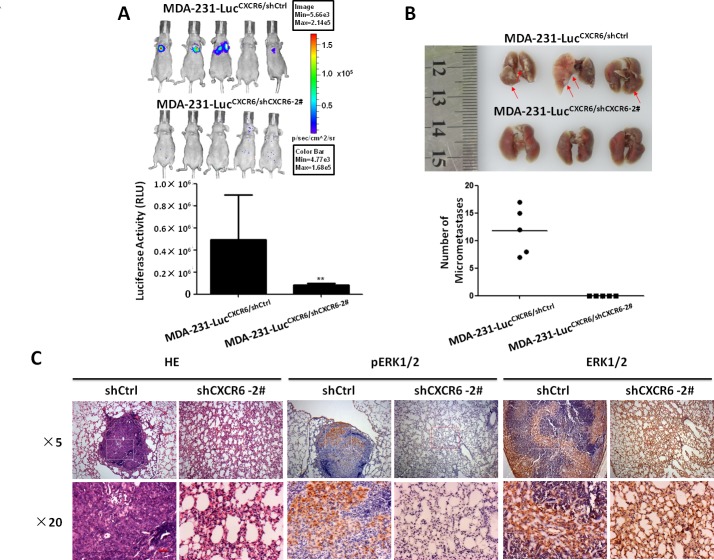
Targeting CXCR6 expression reduces lung metastasis of BC *in vivo* **A**) Targeting CXCR6 expression reduces lung metastasis in transplantable nude balb/c mouse models. Representative bioluminescence images of animals on week 8 are shown. each group n=5, mean ± SEM. **B**) Representative images of lung nodules. MDA-231-luc^CXCR6/shCtrl^ group mice have marked lung metastases as compared with respective MDA-231-luc^CXCR6/shCXCR6-2#^ group mice. Arrows point to the location of metastases. Mean ± SEM. **C**) HE staining of lung tissue and immunohistochemical evaluation of pERK1/2, and ERK in BC tumors grown in nude *balb/c* mice. Luciferase tagged MDA-231^CXCR6/shCtrl^, and respective MDA-231^CXCR6/shCXCR6-2#^ cells were respectively injected into tail vein. After 8 weeks, mice were sacrificed and lungs were harvested and processed for HE staining or immunohistochemistry staining for pERK1/2 and ERK1/2. Original magnification ×10×5 (up) and the white or red frame selected areas were enlarged to magnification ×10×20 (down), where the red bars represents 50 μm.

To further evaluate whether CXCR6 induces tumor metastasis *in vivo*, we transplanted MDA-231-luc^CXCR6/shCXCR6-2#^ and respective MDA-231-luc^CXCR6/shCtrl^ cells tagged with luciferase into nude *balb/c* mice by tail vein injection. The results show that MDA-231-luc^CXCR6/shCXCR6-2#^cells significantly reduced lung metastasis compared with MDA-231-luc^CXCR6/shCtrl^ cells. The bioluminescence imaging noted that MDA-231-luc^CXCR6/shCtrl^ cells formed obviously more lung metastasis compared with MDA-231-luc^CXCR6/shCXCR6-2#^ cells regardless of whether the animal was imaged from ventral surface (Figure 6A) in 8 weeks. Examination of the number of micrometastasis also showed that lung metastasis was markedly decreased in MDA-231-luc^CXCR6/shCXCR6-2#^ mice compared with control mice (Figure 6B). The macroscopic findings were further confirmed by hematoxylin and eosin (H&E) staining (Figure 6C), suggesting that CXCR6 knockdown significantly inhibits BC cells lung metastasis. Additionally, pERK1/2 IHC staining showed that ERK1/2 pathway was significantly suppressed in MDA-231-luc^CXCR6/shCXCR6-2#^ group as compared with MDA-231-luc^CXCR6/shCtrl^, while ERK1/2 expressed no difference in two groups (Figure 6C). These results suggest that reducing CXCR6 expression has a significant effect on inhibiting invasion and metastasis of BC cells by inactivating ERK1/2 pathway.

## DISCUSSION

CXCL16 is a type I membrane protein containing a non-ELR motif-containing CXC chemokine domain in its extracellular region. The cognate receptor for CXCL16 has been identified as CXCR6, a receptor previously shown to be a co-receptor for HIV entry (25, 26). Several groups indicated that CXCL16/CXCR6 signaling correlates with liver-specific homing [30] and lung-specific homing [31, 32] in the events of inflammation [4]. Increasing evidence has shown that CXCL16/CXCR6 chemokine axis plays multifaceted roles in a variety of cancers, including three major aspects of their activities: (1) CXCL16/CXCR6 functions as a regulator in migration and proliferation of a variety of cancer cells[3, 33-35]; (2) CXCR6 functions as a newly defined biomarker of tissue-specific stem cell [16]; (3) regulating angiogenic processes [15]. Our previous studies have demonstrated that CXCL16 signaling through CXCR6 may contribute to prostate cancer progression by serving as a proliferative signal and as a regulator of invasion [11, 36]; however, its role and mechanism of in BC development is still kept unsettled.

In this investigation, we demonstrate that a higher CXCR6 expression in nest site and metastatic lymph node is responsible for BC progression. A similar correlation has been demonstrated in patients with gliomas [37], nasopharyngeal tumors [33], rectal cancer [38], colorectal cancer [39], and melanomas [40], suggesting that CXCR6 expression is important for invasion and metastasis of multiple tumors. Moreover, we found that CXCR6 increased cell migration, invasion and metastasis, associated with increased phosphorylation of ERK1/2 proteins.

Our previous study suggests CXCR6 induces PCa progression by the AKT/mTOR signaling pathway [4]. Whether the ERK pathway is regulated by CXCL16/CXCR6 chemokine axis in PCa cells has not shown in this study. Here, our results show that ERK pathway is activated only after CXCL16 stimulating 5 minutes in both C4-2B^CXCR6^ and LNCaP^CXCR6^ cells. However, this activation is only maintained for a very short period of time (Supplementary Figure 7A). Whether the early and short activations of the ERK pathway induced by CXCL16 stimulation play a role in PCa progression needs further study. Meanwhile, we also detected the AKT/mTOR pathway in BC. The results show that the slight activation of AKT is observed in MCF-7 and MDA-231 cells, but the phosphorylation of P70S6K is rarely changed with CXCL16 treatment in different time pints regardless of CXCR6 expression in MDA-231 cells (Supplementary Figure 7B and 7C). In contrast, the ERK pathway is obviously activated in both MCF-7^CXCR6^ and MDA-231^CXCR6^ cells (Figure 3A, Supplementary Figure 7B and D). These findings suggest the ERK pathway induced by CXCL16/CXCR6 chemokine axis may play more important roles than the AKT/mTOR pathway in BC progression.

In mechanistic analyses, we uncover that RhoA, a member of Rho family GTPase, is involved in these effects. The RhoA, Rac1 and Cdc42 are prototypical members of Rho family representing three canonical sub-groups [41, 42], Different Rho GTPases can produce independent as well as redundant effects [41, 42]. During the process of cell migration, each member of this Rho family GTPase plays a distinct role. RhoA is important for regulating the formation of contractile actin-myosin filaments, which form stress fibers, and for maintaining focal adhesions at the rear of the migrating cells [41, 42].

Accumulating evidence has shown that both cancer invasion and metastasis are directly linked to activation of cofilin, which is regulated by phosphorylation status on Ser3 [43]. Cofilin and ADF (actin-depolymerization factor) are members of a family of essential conserved small actin-binding proteins that play pivotal roles in cytokinesis, endocytosis, embryonic development, stress response and tisuue regeneration [44]. In response to stimuli, cofilin often promotes the regeneration of actin filaments by severing preexisting filaments [45]. The severing activity of cofilin is inhibited by RhoA/ROCK/LIMK phosphorylation at Ser3 [46, 47]. In this investigation, we found that CXCL16/CXCR6 chemokine axis enhances activity of ERK1/2 pathway, which activates RhoA. RhoA activation could lead to actomyosin-based contractility and actin cytoskeletal reorganization through indirectly phosphorylating cofilin and MLC, which could alter the integrity of TJ (tight junction) [23, 24]. Blocking ERK1/2 activation by U0126 apparently decreased the intense expression of actin stress fibers caused by CXCR6 expression. In contrast, reduction of CXCR6 expression in BC cells potentially inhibited the effect. Similar findings were observed in these cells using Y27632 treatment, an inhibitor of the Rho/ROCK pathway. Therefore, CXCR6 expression induces activation of small GTPase RhoA in an ERK1/2 dependent manner. Moreover, it is able to inhibit the activity of cofilin, resulting in enhancing the stability of F-actin, which is associated with the invasiveness and metastasis of BC. To our knowledge, this represents the first report describing the contribution of CXCR6 to activating the ERK1/2/ RhoA/ cofilin /F-actin pathway in BC development. It is important to note that since the ERK1/2 cascade cross-talks with other pathways including AKT (Supplementary Figure. 6C), combinations of ERK1/2 pathway inhibitors with other therapeutic agents have produced synergistic effects on inhibiting tumor growth in experimental models and in some clinical trials [48, 49]. The immediate challenge is therefore to determine how ERK1/2 pathway inhibitors can be applied in a tumor-specific manner to minimize adverse effects by blocking a pathway with broad biologic significance.

In addition, we found that CXCR6-overexpressing BC cells acquired polar cell morphology and exhibited the enhanced cellular motility (40). It suggests that alteration of CXCR6 may induce Epithelial-mesenchymal transition (EMT) in the process of BC progression. However, our results show that overexpression of CXCR6 and then treatment with CXCL16 for 24 or 48 hours in BC cells didn't alter the apparent expression of E-cadherin epithelial markers and vimentin mesenchymal markers (Supplementary Figure 3). These results suggest that CXCL16/CXCR6 chemokine axis may have no function on EMT in the process of BC progression. Cell morphological changes may be due to the changes of cytoskeletal system mediated by F-actin rearrangements.

*In vivo* data show CXCR6 knockdown significantly reduced BC cells lung metastasis. In line with the finding, IHC stainings show the ERK1/2 pathway was significantly suppressed in lung metastases, suggesting that ERK1/2 pathway may play a key role in CXCR6- promoted BC cells distant metastases.

**Figure 7:**
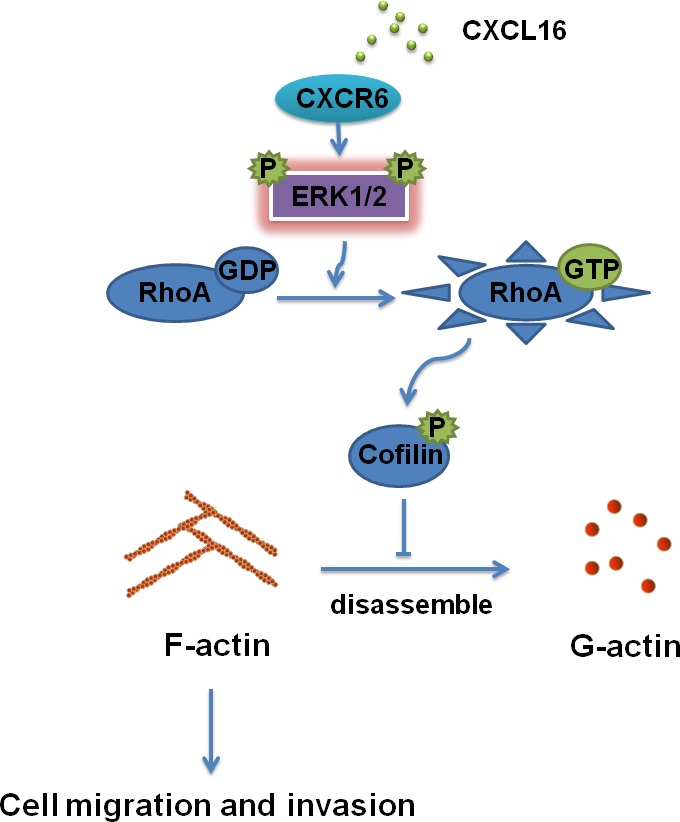
Schematic diagram showing a novel regulatory mechanism for CXCL16/CXCR6 chemokine axis -induced BC progression CXCL16/CXCR6 chemokine axis modulates activation of RhoA through activating ERK1/2 signaling pathway, which inhibits the activity of cofilin, thereby enhancing the stability of F-actin, responsible for BC invasiveness and metastasis.

Overall, our data demonstrate a novel mechanism underlying the CXCR6 / ERK1/2/ RhoA / cofilin /F-actin pathway in the development of BC as shown a working model in Figure 7. Blocking the signaling pathway may provide a more effective therapeutic strategy for BC.

## MATERIALS AND METHODS

### Cell cultures

The human breast cancer cell lines MCF-7, MDA-231, MDA-468, MDA-435, MDA-453 and SK-BR-3 were obtained from the American Type Culture Collection and cultured according to the online instructions of the manufacturer. The immortalized epithelial cell line HBL-100 (Cell Bank of Shanghai Institute for Biological Sciences, Shanghai, China) was maintained in DMEM (Hyclone) supplemented with 10% FBS (GIBCO). The human prostate cancer (PCa) cell line LNCaP was purchased from the American Type Culture Collection. The metastatic subline LNCaP C4-2B (C4-2B) cells were originally isolated from a lymph node of a PCa patient with disseminated bone and lymph node involvement. LNCaP and C4-2B cells were maintained at 37°C in an atmosphere of 5% CO_2_ in an RPMI-1640 medium supplemented 10% FBS. All BC (MCF-7^CXCR6^, MDA-231^CXCR6^) and PCa (C4-2B^CXCR6^, LNCaP^CXCR6^) cell lines stably expressing CXCR6 and the respective GFP-expressing control cells were cultivated in DMEM or RPMI-1640 (Corning Cellgro) supplemented with 100 μg/ml G418. MDA-231-luc^CXCR6/shCtrl^ and MDA-231-luc^CXCR6/shCXCR6-2#^ cells were cultivated in DMEM supplemented with 0.5 μg/ml puromysin and 100 μg/ml G418. All wild-type cell lines were tested and authenticated by DNA typing in the Shanghai JiaoTong University Analysis Core (last test in April 2013). Cells were cultured in a humidified environment containing 5% CO_2_ and held at a constant temperature of 37°C.

### Detection of CXCR6 expression by flow cytometry

Cells cultivated in 100 mm dishes were digested by 0.25% trypsin (supplemented with 0.5mM EDTA). After washed with PBS for 3 times, cells then were resuspended in an isotonic PBS buffer to a final concentration of 4 × 10^6^ cells/ml and 25 μl of cells were transferred to a 5 ml tube for CXCR6 staining. In brief, after Fc-blocked by IgG, cells were cultivated with APC-conjugated anti-CXCR6 reagent (R&D, CAT: FAB699A). Then resuspend the cells in PBS buffer for flow cytometric analysis. Details are show in the supplementary materials and methods.

### Active RhoA pull-down assays

The assays were performed with the Active Rho Pull-Down and Detection kit (CAT#: 0016116, Thermo Fisher Scientific) according to the manufacture's instruction. In brief, cells were lysed by 1×lysis buffer and the same amount of total proteins was incubated with agarose beads and GST-Rhotekin-RBD protein-binding domain. After incubation at 4°C for 1 h, the samples were washed three times with 0.4 ml of 1× lysis buffer. Following the last wash, 50 μl 2× reducing buffer was added to the samples and incubated at room temperature for 2 min. At last the samples were collected by centrifugation at 6000g for 2 min and were electrophoresed on an SDS-PAGE gel followed by western blot analysis.

### Stress fiber analysis

Cancer cells of ~30% confluence were starved for 30 min, and then treated with DMSO or inhibitors (2 μM U0126 (CAT: S1102, Selleck), 10 μM Y27632 (CAT: S1049, Selleck), 10 μM PF573228 (CAT: S2013, Selleck)) for 30 min following treated with or without 100 ng/ml rhCXCL16 for additional 5min, and then the cells were fixed with PBS containing 4% paraformaldehyde without methanol. Then cells were stained with rhodamine phalloidin according to the manufacturer's protocol (Invitrogen). More than 25 individual cells per treatment group were visualized by confocal microscopy (LSM-710META, Carl Zeiss) in each experiment. The numbers of stress fibers in each cell was quantitated as previously described [27]. Briefly, the Image Pro Plus software (Media Cybernetics, Bethesda, MD, USA) was used to generate line profiles for each cell. A graphic depiction was then generated where the x axis represented the distance across the cell, the y axis represented the level of fluorescence and each immunofluorescence intensity spike represented an individual stress fiber crossed by the line. The fluorescence intensity level of 100 was set as the cutoff of stress fiber spike, as the background intensities outside cells were never greater than this value.

### Tissue microarray and immunohistochemistry

The immunohistochemical analysis was performed using the avidin-biotin-peroxidase complex method with an anti-CXCR6, anti-pERK1/2 or anti-ERK1/2 antibody. Details are provided in the Supplementary materials and methods section.

### Construction of CXCR6 stably overexpression cells

The full-length human CXCR6 cDNA were amplified from MDA-231 cells by reverse transcription-PCR and inserted into the lentivirus vector pLenti-easy-HA. Lenti-x cells were then transfected with the pMD2.G, psPAX2 and pLenti-easy-CXCR6 expression vectors using Lipofectamine 2000 (Invitrogen). After 48 h, culture supernatants were collected, passed through 0.45 μm filters, and mixed with fresh media (1:1) and polybrene (8 μg/ml) to infect target cells. Cells with restored expression of CXCR6 were designated as MCF-7^CXCR6^, MDA-231^CXCR6^, C4-2B^CXCR6^ and LNCaP^CXCR6^, the respective controls cells infected with pLenti-easy-GFP lentiviral vectors were designated as MCF-7^GFP^, MDA-231^GFP^, C4-2B^GFP^ and LNCaP^GFP^. Then the CXCR6 positive cells and GFP control cells were sorted by flow cytometry. Stable cell lines were established using G418 selection.

### Construction of RhoA and mutated RhoA plasmids

RhoA cDNA was amplified from MDA-231 cells and were inserted into p3×FLAG-CMV-14 plasmid vector, and the RhoA^V14^, RhoA^N19^ mutation plasmids were constructed using KOD-Plus- Mutagenesis Kit (CAT: KOD-201, TOYOBO) according to the manufacturer protocal. In brief, firstly, the mutation sites were designed to N terminal of forward primer, and then use wild-type RhoA-p3×FLAG-CMV-14 plasmid as a template for reverse PCR. Secondly, digest the template plasmid by Dpn I enzyme. Thirdly, perform self-ligation of the PCR product by T4 polynucleotide kinase. Finally, Use E. coli DH5α as the transformation host for plasmids transformation. All primers are shown in supplementary Table 2.

### Western blot analysis

The expression of proteins was evaluated using western blot analysis. In brief, 2.5-5.0×10^5^ cells in 6-well plates were incubated with 100 ng/ml of rhCXCL16 (CAT#: 976-CX-025, R&D) pretreated with or without ROCK inhibitor Y-27632, the MAPK/ERK kinase 1 (MEK1) inhibitor U0126 for the designated time points. The cells were then washed in PBS and suspended in 100 μl of RIPA buffer (Pierce). Supernatant protein concentrations were determined using the BCA protein assay kit (Pierce). Supernatant samples containing 30μg total protein were resolved by 10% or 12.5% SDS–PAGE gels depending on the sizes of target proteins, transferred to Immobilon-P PVDF membranes (Millipore, Billerica, MA) by electroblotting, and then probed with anti-ERK1/2, anti-phospho-ERK1/2, anti-Cofilin, anti-phospho-Cofilin, anti-P70S6K, anti-phospho-P70S6K, anti-GAPDH, anti-RhoA, anti-E-adherin, anti-vimentin (Cell Signaling Technology), anti-CXCR6 antibodies (Epitomics). Membranes were incubated with horseradish peroxidase-conjugated secondary antibodies. Blots were developed using an ECL kit (Millipore).

### Migration and invasion assays

A wound healing assay was used to assess cell migration ability and cell invasion was examined using a reconstituted extracellular matrix membrane (CAT: 354483, BD, USA). The procedures are as our previously described [28].

### shRNA transfection

Lenti-x cells were transfected with the pMD2.G, psPAX2 and shRNA-CXCR6 plasmids or shRNA vectors (GV248 lentiviral vectors) using Lipofectamine 2000 (Invitrogen). After 48 h, culture supernatants were collected, passed through 0.45 μm filters, and mixed with fresh media (1:1) and polybrene (8 μg/ml) to infect MDA-231^CXCR6^ cells. Cells infected with three different shRNA-CXCR6 or control vectors were respectively designated as MDA-231^CXCR6/shCXCR6-1#, -2# and -3#^ and MDA-231^CXCR6/shCtrl^. Stable cell lines were established using 1 μg/ml puromysin selection. The seed sequences of shRNA-CXCR6-1#, -2# and-3# as follows: 1#: 5′-CTGAGGACAATTCCAAGACTT-3′, 2#: 5′-CTCACCATGATTGTCTGCTAT-3′, 3#: 5′-GCTTGCTCATCTGGGTGATAT-3′.

### Metastasis assay *in vivo*

The modified transplantation through tail vein injection is referred to in the report of Zheng et al [29]. Briefly, Tail vein injections were performed to determine whether knockdown of CXCR6 expression played a role in metastasis. Using a 1 ml syringe fitted with a 25-gauge needle, 5- to 6-week-old nude *balb/c* mice (Slaccas Laboratory Animal, Shanghai, China) were injected into tail vein with MDA-231-luc^CXCR6/shCXCR6-2#^ and respective MDA-231-luc^CXCR6/shCtrl^ cells (1× 10^6^/0.1 ml/mouse). After 8 weeks, bioluminescence was utilized to follow the BC cell-derived tissue metastases. The mice were injected intraperitoneally with 200 μl of 15 mg/ml luciferin prior to the Xenogen IVIS cryogenically cooled imaging system to detect the tumor metastasis.

### Statistical analysis

Statistical analyses were performed using SPSS 16.0. Differences among variables were analyzed by 2-tailed Student's t tests. Data were presented as the mean ± SD unless otherwise indicated. Chi-square test was used to analyze the CXCR6 expression differences in tissue microarray. Statistical significance was defined as *P* < 0.05.

## SUPPLEMENTARY MATERIALS AND METHODS, FIGURES AND TABLES


