# Supplementary material for: CXCL16/CXCR6 chemokine signaling mediates breast cancer progression by pERK1/2-dependent mechanisms
Source: Oncotarget. 2015 Mar 29;6(16):14165–78. doi: 10.18632/oncotarget.3690 (PMC4546458; doi:10.18632/oncotarget.3690)
Supplement: Supplementary file 1 [file oncotarget-06-14165-s001.pdf]

CXCL16/CXCR6 chemokine signaling mediates breast cancer progression by pERK1/2-dependent mechanisms

Supplementary materials and methods

Detection of CXCR6 expression by flow cytometry

Cells cultivated in 100 mm dishes were digested by 0.25% trypsin (supplemented with 0.5mM EDTA). After washed with PBS for 3 times, cells then were resuspended in an isotonic PBS buffer to a final concentration of 4×10^6 cells/ml and 25 µl of cells transferred to a 5 ml tube for CXCR6 staining. Cells were Fc-blocked by treatment with 1 µg of human IgG/10^5 cells for 15 minutes at room temperature prior to staining. Then transfer 25 µl of the Fc-blocked cells to a 5 ml tube, add 10 µl of APC-conjugated anti-CXCR6 reagent (R&D, Cat: FAB699A). Incubate for 30 - 45 minutes at 2-8℃. Following this incubation, remove unreacted anti-CXCR6 reagent by washing the cells twice in 4ml of the same PBS buffer. Resuspend the cells in 200 – 400 µl of PBS buffer for flow cytometric analysis. For CXCR6 positive cell sorting assay, the APC positive cells were re-collected in a new 5 ml tube.

Tissue microarray and Immunohistochemistry

High-density tissue microarrays were constructed by US Biomax Inc using clinical samples obtained from a cohort of 192 patients including 80 cases of each invasive ductal carcinoma and invasive lobular carcinoma, 32 adjacent normal tissue or normal tissue, single core per case (Cat: BR1921a, US Biomax, USA). Paraffin-embedded tissue sections (4 µm) were deparaffinized by xylene and rehydrated in a graded alcohol series (100%, 95%, 80% and 70%, 5 min each). After antigen retrieval in sodium citrate buffer (10 mM, pH=6.0), endogenous peroxidase was blocked by 0.3% H_2O_2 in methanol for 10 min. Tissue sections were blocked with normal goat serum (NGS, 5%) for 30 min at room temperature and then incubated with rabbit anti-human CXCR6 (1:50, Cat: ab8023, Abcam), anti-human pERK1/2 (1:100, Cat:4376S, CST) or anti ERK1/2 (1:200, Cat:9102S, CST) antibody at 4°C overnight. After washing (1×PBS, three times, 5 min each), tissue sections were

incubated with biotinylated goat anti-rabbit antibody (1:200, Vector Laboratories, Burlingame, USA) at room temperature for 30 min. Then, tissue sections were incubated with the avidin-biotin-complex-PO (Vector Laboratories, California, USA) and developed in DAB Coloring Agent (Sigma, St Louis, MO, USA). Negative controls were generated by omission of the primary antibody. The staining intensity was scored on a scale of 5 as follows: negative (-), negligible (±), weak (+), moderate (++) and strong (+++). Negative (-), negligible (±) and weak (+) were defined as low level expression of CXCR6, and the moderate (++) and strong (+++) respectively defined as median and high expression. Images were obtained using a microscope (Zeiss) with a 10× objective lens. Chi-square test was used to analyze the CXCR6 expression differences in different tissues.

Real-Time PCR

Total RNA was extracted by Trizol reagent (Ambion, Cat: 15596018) according to the manufacturer's instructions. Less than 2 ug RNA was reverse-transcripted into cDNA using reverse transcript kit (Takara, Cat: RR036A). Primers we are showed in supplementary table 3. PCR was performed with an ABI 7500 Fast Real-Time PCR System using SYBR Premix Ex Taq™ (TLi RNaseH Plus) (Takara, Cat: RR420A). PCR protocol was performed as follows: denaturing for 3 seconds at 95 ℃ followed by 40 ampliication cycles of annealing and extension at 60 ℃ for 30 seconds. After normalized to GAPDH gene, expression levels for each target gene were calculated using the comparative threshold cycle (CT) method. The △Ct values were calculated according to the formula △Ct = Ct (gene of interest) - Ct (GAPDH) in correlation analysis, and the $2^{-\triangle\triangle Ct}$ was calculated according to the formula $2^{-\triangle\triangle Ct} = 2^{-(\triangle Ct\ (experimental\ group)\ -\ \triangle Ct\ (control\ group))}$ for determination of relative. Data are presented as the mean ± standard deviation (SD) from three independent experiments.

Supplementary Table 1.

Statistical and quantitative analysis of CXCR6 expression in tissue microarray. Chi-square test was used to analyze the CXCR6 expression differences in different tissues.

			CXCR6_Expression_Levels			Total	P-Value
			low	median	high		
Type	Normal	Count	31	1	0	32	.000
		% within Type	96.9%	3.1%	.0%	100.0%	
	Tumor	Count	1	62	96	159	
		% within Type	.6%	39.0%	60.4%	100.0%	
Total		Count	32	63	96	191	
		% within Type	16.8%	33.0%	50.3%	100.0%	

Supplementary Table 2.

Vectors and primers used in plasmids construction.

Gene	vector	Forward primer	Reverse primer
CXCR6	Lenti-easy-HA	TAGGGGTACCGCCGCCACCATGGCAGAGCATGATTACCATG	ATTGGGATCCTAACTGGAACATGCTGGTGGCC
RhoA	p3×Flag-CMV-14	TATAGGTACCGCCGCCACCATGGCTGCCATCCGGAAGAAAC	AATTGGATCCCAAGACAAGGCACCCAGATT
RhoA^{V14}	pMSCVpuro	GTAGCCTGTGGAAAGACATGCTTGCTCA	ATCACCAACAATCACCAGTTTCTTC
RhoA^{N19}	pMSCVpuro	AACTGCTTGCTCATAGTCTTCAGCAAGG	CTTTCCACAGGCTCCATCACCAACA

Supplementary table 3.

Primers used in the Real-Time PCR.

Gene	Forward primer	Reverse primer
MYT1	ACACCTCTCACACCTCTG	CTCGTCCTTGTCATCATCC
KIF6	CGATGTCTGTGATGTGAATG	TTGATGAAGGCGATGATGT
MAPT	CCACTGAGAACCTGAAGC	TTGCCTAATGAGCCACAC
Trim29	GCTGTGGACCAAGTGAAG	GAGTCGCTGATGCTATGC
CAV1	GAGCGAGAAGCAAGTGTA	ACAGTGAAGGTGGTGAAG
FRY	GAGCCGTTCTTCCTTCAG	CAGCATCATACACCTTCCTA
TNS4	ACCTTGACTCCTACATTGAC	GGCTTCAGATTCCTCCTTC
COL21A1	TGCCAAGTCCTCACGAT	CTGAACCAACACCAATAGC
MXRA5	GATGGCACTCTCCTTATTCA	AATCCACACCGTCTTCCT
DEF6	ATGAAGAATCTGTGCGAATC	CTCTGCTGTGCCATCTC
ZC3H12D	CTACGACGACCGCTACAT	GCTCAGGAAGTTGCTCAG
ATCG2	GTGCGAGACATCAAGGAG	AAGGAAGGCTGGAAGAGG
AJAP1	CTGCTCTCATCACAACTCT	CTCTCCTCCTGCTGGTT
SVEP1	GTCTCCTGCTACACATTCA	CTGCCATTCCAAGTTCCA
CXCR6	CAGGAGGAGCATCAAGAC	GGATATGACCAGCACCAG
GAPDH	ACGGATTTGGTCGTATTGGG	CGCTCCTGGAAGATGGTGAT

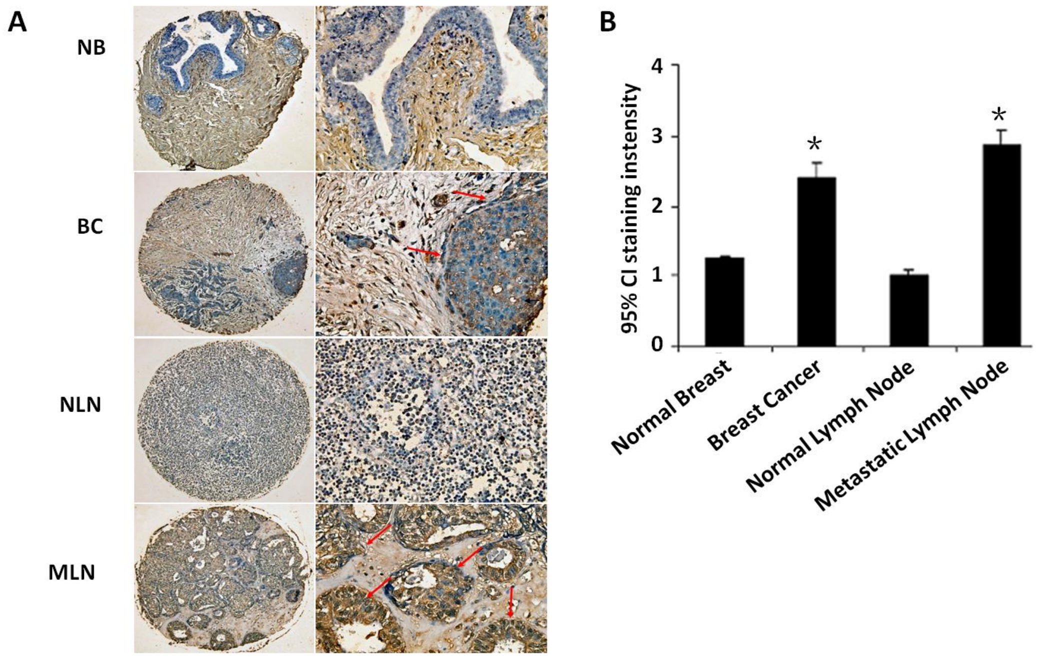

Supplementary Figure 1

CXCR6 expression in human breast cancer tissue microarray

A) immunohistochemical evaluation of CXCR6 in a human breast cancer tissue microarray（Cat： ARY-HH0058， Columbus, OH). Representative microscopic images of normal breast (NB), breast cancer (BC), normal lymph node (NLN) and metastatic lymph node (MLN) tissues stained with an anti-human CXCR6 antibody. The right pictures are the enlarged diagrams of their corresponding left pictures.

B) Chi-square test was used to analyze the CXCR6 expression differences in different tissues.

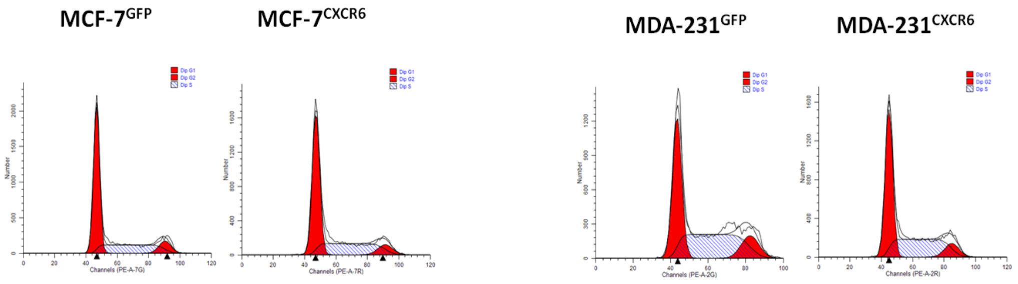

Supplementary Figure 2

CXCR6 has no effect on the cell cycle distribution. 1×10^6 MCF-7^{CXCR6}, MDA-231^{CXCR6} and their respective control cells were collected for cell cycle analyses by FACS, both MCF-7 and MDA-231 cell pairs show a similar cell cycle distribution.

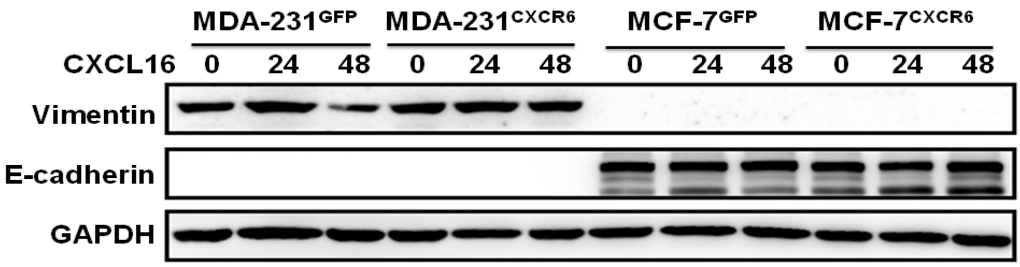

Supplementary Figure 3

The role of EMT by CXCL16/CXCR6 chemokine axis in breast cancer cell

CXCL16/CXCR6 chemokine axis has no effect on the expressions of E-cadherin and Vimentin in MDA-231^{CXCR6}, MCF-7^{CXCR6} and their respective control cells. Cells treated with CXCL16 (100 ng/ml) for indicated time points, expressions of E-cadherin and Vimentin were analyzed by western blot.

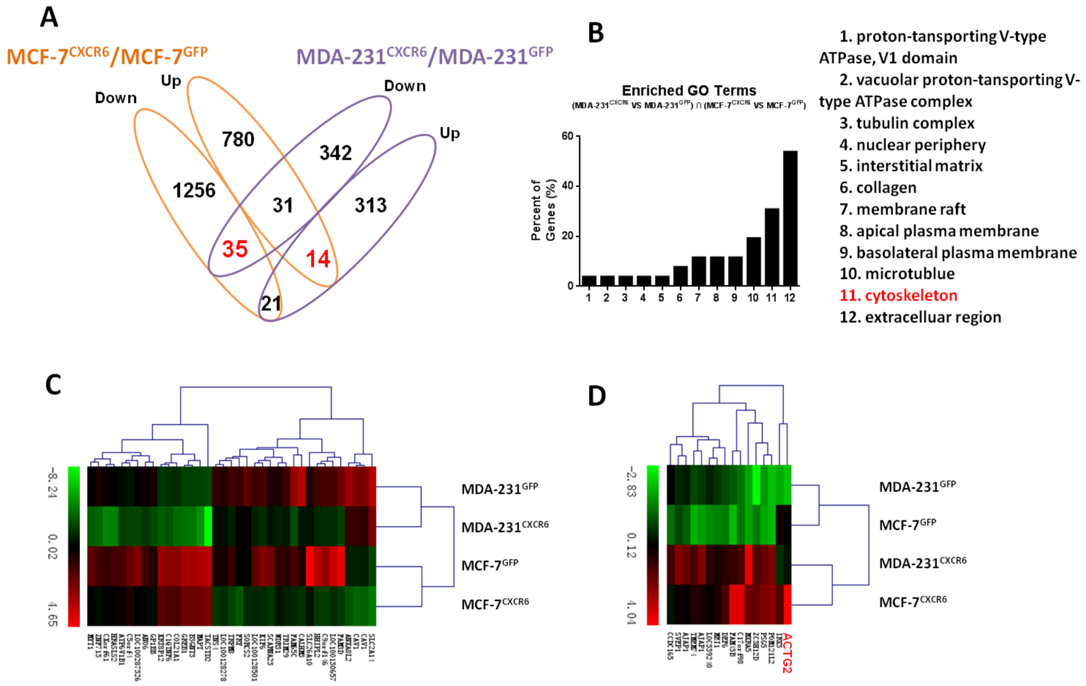

Supplementary Figure 4

CXCR6 modulates cytoskeleton in breast cancer cells.

A) Venn diagram shows the overlap of differentially expressed genes (fold change ≥2) in the two cell groups.

B) Significant GO terms of differentially expressed genes. A bar plot is generated from the top 12 GO containing the largest fold enrichment of differentially expressed genes. The plot gives a more intuitive view of the significant GO terms. The cytoskeleton is the secondly largest fold enrichment GO (Red font).

C) Clustering map of 35 down regulated genes.

D) Clustering map of 14 up regulated genes. Columns represent genes; rows represent experimental cells. ACTG2 (Red font) as an F-actin monomer protein gene was significantly unregulated in MCF-7^{CXCR6} and MDA-231^{CXCR6} cells compared with their respective control cells.

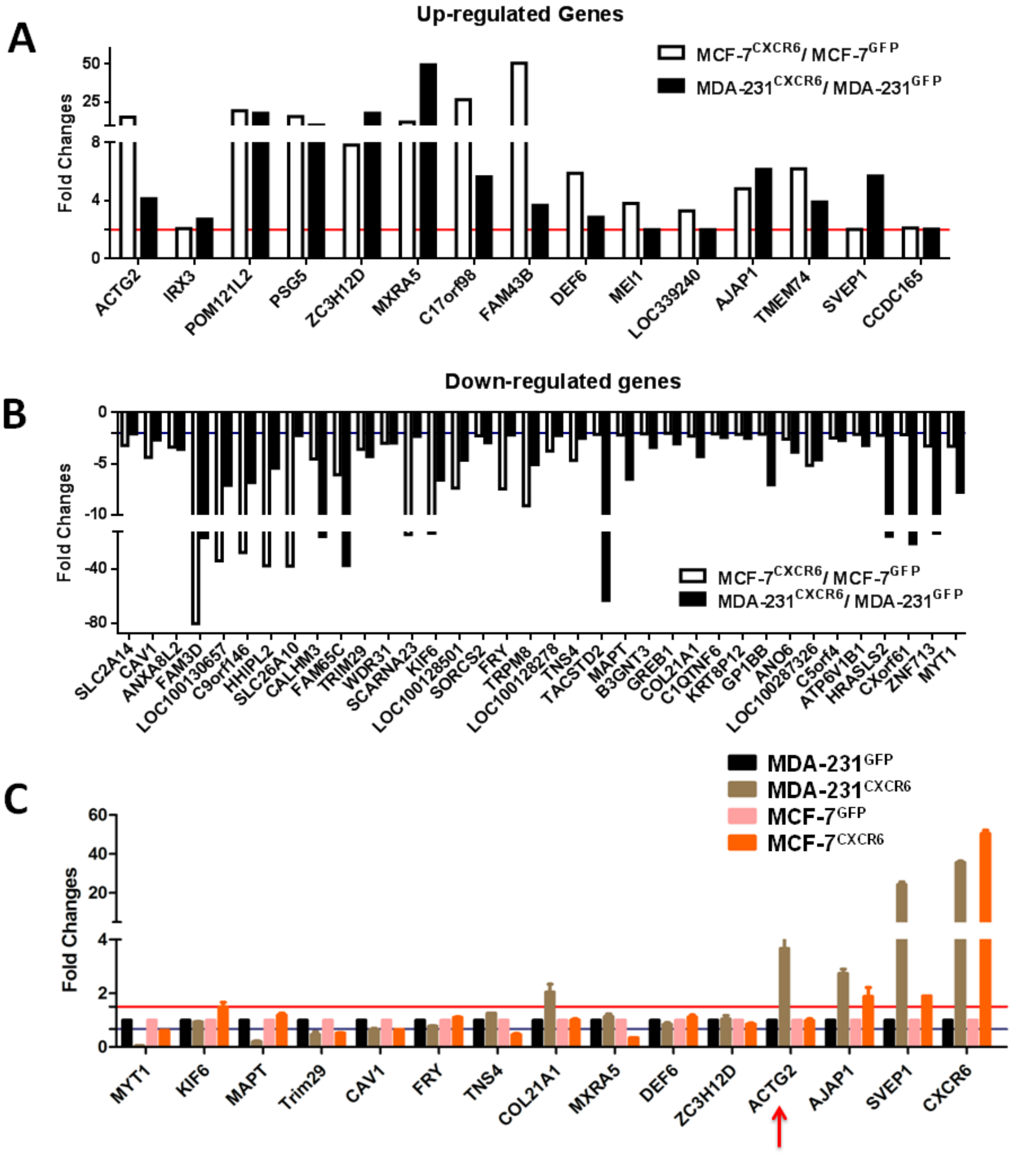

Supplementary Figure 5

Genes changed more than 2-fold regulated by CXCR6 overexpression in breast cancer cells.

A) and **B)** respectively shows the fold chhanges of 14 up-regulated and 35 down-regulated genes analyzed by Agilent Whole Human Genome Microarrays. The red line represents upregulated 2-fold, the blue line represents downregulated 2-fold.

C) Real-time PCR was used to further confirm the change trends of interesting genes showed in A) or B). The red line represents upregulated 1.5-fold, the blue line represents downregulated 1.5-fold.

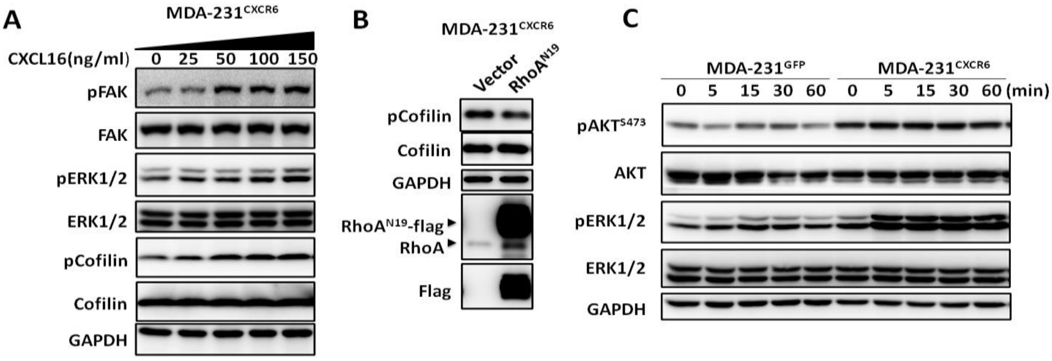

Supplementary Figure 6

CXCL16/CXCR6 chemokine axis activates the ERK1/2, AKT and FAK pathways and inhibits cofilin activity.

A) CXCL16 significantly activates the pERK1/2 pathway in MDA-231^{CXCR6} cells, but not in MDA-231^{GFP} cells. However, the activity of the AKT pathway is higher in MDA-231^{CXCR6} than MDA-231^{GFP} cells whether treatment with CXCL16 or not.

B) MDA-231^{CXCR6} cells exhibit phosphorylation of FAK, ERK and cofilin in response to CXCL16 stimulation in dose-dependent manner. Cells treatment with 100ng/ml of CXCL16 for 5 minutes, the phosphorylation status of FAK, ERK and cofilin were detected by western blot analysis.

C) DN-RhoA^{N29} promotes cofilin activity by reducing the phosphorylation of cofilin in MDA-231^{CXCR6} cells. MDA-231^{CXCR6} cells transfected with flag-tagged RhoA^{N19} or vector plasmids for 24 hours, the phosphorylation status of cofilin was detected by western blot analysis.

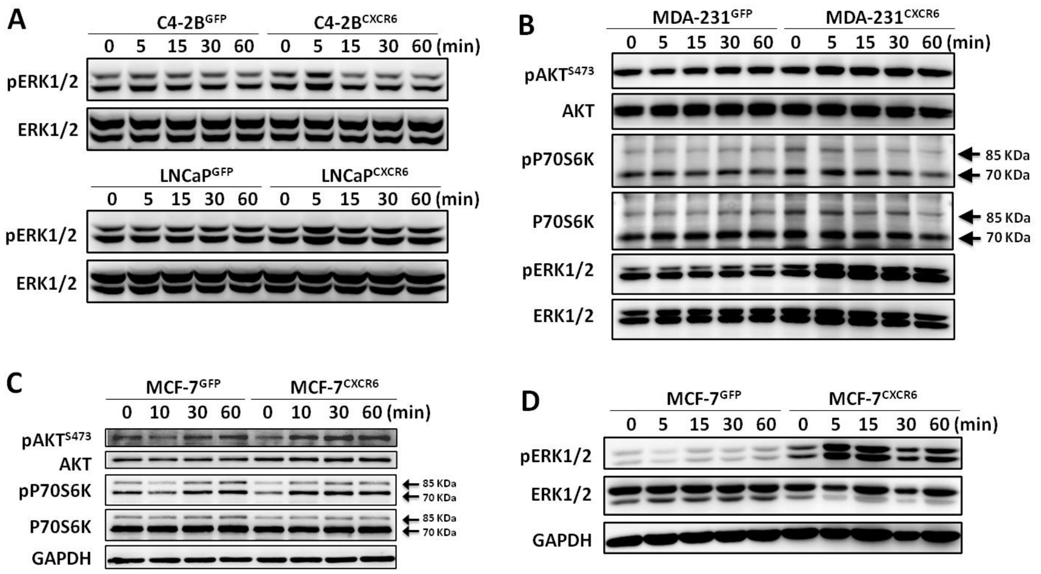

Supplementary Figure 7 Legend

The AKT/mTOR and ERK1/2 pathways are induced by CXCL16/CXCR6 chemokine axis in breast cancer and prostate cancer cells.

A) and D) The ERK1/2 pathway was detected in C4-2B^{CXCR6}, LNCaP^{CXCR6} and MCF-7^{CXCR6} and their respective control cells after treatment with 100 ng/ml CXCL16 for the indicated times.

B) The AKT/mTOR and ERK1/2 pathways were detected in MDA-231^{CXCR6} and MDA-231^{GFP} cells after treatment with 100 ng/ml CXCL16 for the indicated times.

C) The AKT/mTOR pathway was detected in MCF-7^{CXCR6} and MCF-7^{GFP} cells after treatment with 100 ng/ml CXCL16 for the indicated times.

For **B)** and **D)**, the representative figures show that after detected the phosphorylation status of AKT, P70S6K or ERK1/2, the PVDF membranes were stripped and used for their respective total protein detection.
